# Biological Control of *Aspergillus parasiticus* and *Aspergillus ochraceus* and Reductions in the Amount of Ochratoxin A and Aflatoxins in Bread by Selected Non-Conventional Yeast

**DOI:** 10.3390/foods12203871

**Published:** 2023-10-22

**Authors:** Izabela Podgórska-Kryszczuk, Urszula Pankiewicz, Lidia Sas-Paszt

**Affiliations:** 1 Department of Analysis and Food Quality Assessment, University of Life Sciences in Lublin, Skromna 8, 20-704 Lublin, Poland; urszula.pankiewicz@up.lublin.pl; 2 Department of Microbiology and Rhizosphere, The National Institute of Horticultural Research, Konstytucji 3 Maja 1/3, 96-100 Skierniewice, Poland; lidia.sas@inhort.pl

**Keywords:** *Aspergillus parasiticus*, *Aspergillus ochraceus*, non-conventional yeast, biocontrol, ochratoxin A, aflatoxins

## Abstract

*Aspergillus parasiticus* and *Aspergillus ochraceus* are important pathogenic fungi that pose a serious threat because of their ability to produce mycotoxins, including ochratoxin A (OTA) and aflatoxins (AFs). The main method of reducing these pathogens is the use of chemical fungicides, though recently there has been a focus on finding biological control agents. The obtained results from this study indicate the great potential of two wild yeast strains, *Aureobasidium pullulans* PP3 and *Saitozyma podzolicus* D10, in the biological control of *A. parasiticus* and *A. ochraceus* and reductions in the amount of OTA and AFs they produce. In vitro, the growth of the mycelium of pathogens was reduced by 41.21% to 53.64%, and spore germination was inhibited by 58.39% to 71.22%. Both yeast strains produced the enzymes chitinase, β-1,3-glucanase, and amylase, and *A. pullulans* PP3 additionally produced protease and cellulase. This yeast strain also had the ability to grow over a wide range of temperature (4–30 °C), salinity (0–12%) and pH (4–11) conditions. No growth of the yeast was observed at 37 °C, nor any biogenic amines or hydrogen sulfide production. Adding the tested yeast inoculum to the dough reduced OTA (within 14.55–21.80%) and AFs (within 18.10–25.02%) in the model bread.

## 1. Introduction

Filamentous fungi and the toxic metabolites they produce (mycotoxins) significantly reduce food and feed quality and pose a significant threat to food safety. Mycotoxins are low-molecular and thermostable substances resistant to most technological processes, such as cooking, baking, frying, distillation, and fermentation [1]. The main source of human exposure to mycotoxins is food. These compounds enter the human body through two ways: (1) primary—from contaminated food products of plant origin; and (2) secondary—through contaminated animal tissues (meat, milk, and eggs) from animals fed with contaminated feed [2]. Even low levels of mycotoxins in food may result in serious health consequences [1]. Fungi are common in the environment due to their ability to grow on almost any substrate and under harsh conditions [3]. The *Aspergillus* spp. include a range of filamentous fungi occurring in various ecological niches around the world and can cause health- and life-threatening diseases, especially in immunocompromised individuals [4]. The most dangerous species in this genus include *A. flavus*, *A. parasiticus* [5], *A. ochraceus*, and *A. carbonarius* [6]. These fungi produce highly harmful secondary metabolites, including aflatoxins (AFs) and ochratoxin A (OTA) [7]. The most toxic and carcinogenic naturally occurring toxins are AFs. They cause teratogenic, carcinogenic, mutagenic, and immunomodulatory changes in humans and animals [8]. Products contaminated with AFs are mainly cereals, cereal products, dried fruits, nuts, and beer. OTA is also found in various foods, mainly cereals and grain products [7]. This toxin exhibits teratogenic, carcinogenic, immunotoxic, hepatotoxic, genotoxic, and neurotoxic effects [9]. Contamination of crops with fungi and mycotoxins is a crucial threat to food and feed safety and has serious economic consequences and impacts for international trading [10].

The most common method of controlling pathogenic fungi is by chemical means, i.e., fungicides. However, they are not indifferent to consumer health and the natural environment [11]. Fungicides often pose a serious threat to non-target organisms. Due to the widespread use of these substances, as well as accumulation both in the human body and in the environment, fungicides are found in all elements of the environment, including the air, soil, water, bottom sediments and even plants, animal organisms, and food. Another serious threat from chemical pesticides is the resistance of pathogens to these substances [12]. The environmental impact of agriculture is becoming increasingly analyzed, and reducing the climate and environmental footprint of the European Union’s food system is part of the EU Green Deal. As part of this, the European Commission proposes reducing the use and risk of chemical pesticides by 50% by 2030 [11]. Therefore, interest in biological methods of fungal control, including antagonistic microorganisms, has increased. A great number of microorganisms have been identified worldwide that exhibit antagonistic effects against pathogenic fungi. One group of antagonistic microorganisms used in biocontrol that is becoming increasingly popular among scientists and industry is yeasts. Among them are selected species mainly from the following genera: *Aureobasidium*, *Candida*, *Debaryomyces*, *Metschnikowia*, *Pichia*, *Pseudozyma*, *Saccharomyces*, and *Tilletiopsis* [13]. Yeast uses a variety of mechanisms to prevent the development of pathogenic fungi during both growth and storage [3]. Yeast’s main mechanisms of action essential in the biological control of fungi are the following: (1) competition for nutrients and space; (2) the secretion of lytic enzymes degrading the fungal cell wall; (3) the production of volatile organic compounds (VOCs); (4) the secretion and release of antimicrobial compounds such as killer toxins or “mycocins”; and (5) tolerance to high concentrations of ethanol [14]. Yeast cells and their metabolites have great potential in minimizing economic losses caused by pathogenic fungi. Many species have proven antimicrobial activity [15] and can reduce mycotoxins [16,17,18]. There are now great hopes for developing new antimicrobial starter cultures that will reduce the occurrence of fungi in food and reduce the amount of toxins they produce, thus improving food quality and safety. To this day, such starter cultures mainly consist of lactic acid bacteria [19], but several yeast strains also have great potential, including *Sachcaromyces cerevisiae* in the baking industry [20] or *Debaryomyces hansenii* in meat production [21].

This study aimed to select, identify, preliminarily characterize, and investigate the efficacy of yeast isolates showing inhibition potential from two fungal species, *A. parasiticus* and *A. ochraceus*, and test their ability to reduce AFs and OTAs in bread.

## 2. Materials and Methods

### 2.1. Microorganisms

The study used fungi, *A. parasiticus* KKP731 and *A. ochraceus* KKP439, and 51 morphologically diverse wild yeast strains: 8 from the Symbio Bank located in the Department of Microbiology and Rhizosphere, the National Institute of Horticultural Research in Skierniewice (Poland), and 43 isolated from ears of wheat that were obtained from a conventional farming field located in the Lublin Voivodeship (eastern Poland). The viability of pure cultures was maintained throughout the study by periodic transfer to Wort agar (BTL, Lodz, Poland) under aseptic conditions. Microorganisms were kept at 4 °C for routine cultivation.

#### Yeast Isolation and Identification

To isolate the yeast from the environment, 5 g of wheat ears was placed in flasks with 45 mL of sterile Ringer’s solution. Then, the flasks were placed on a rotary shaker at 180 rpm for 10 min. After this time, serial dilutions of the obtained suspensions were made and transferred to Petri dishes with yeast glucose chloramphenicol agar (BTL, Lodz, Poland). After 48 h of 28 °C incubation, single yeast and yeast-like fungi colonies were transferred to Wort agar (BTL, Lodz, Poland).

Only selected yeast isolates that most effectively limited the growth of *A. parasiticus* KKP731 and *A. ochraceus* KKP439 in the preliminary efficacy assessment test (confrontation assay) were genetically identified. Genetic identification of selected yeast isolates was performed according to the procedure of the Nexbio laboratory (Nexbio Sp. z o. o., Lublin, Poland) using the universal primers ITS1 (5′-CTTGGTCATTTAGAGGAAGTAA-3′) and ITS4 (50-TCCTCCGCTTATGATGC-30).

### 2.2. Confrontation Assay

Inhibition of *A. parasiticus* KKP731 and *A. ochraceus* KKP439 mycelial growth by the yeasts was evaluated in Petri dishes with 20 mL of Wort agar (BTL, Lodz, Poland) according to the methodology described earlier [3]. A 5 mm in diameter pathogen mycelium disc sterilely cut from a 7-day-old culture was placed 3 cm from the edge of the dish. Next, at 3 cm from the opposite edge, Petri dishes were inoculated by linear streaking with a loop of 2-day-old yeast culture. The dish inoculated with only fungal pathogens was a control. The Petri dishes were incubated for 7 days at 28 °C. After this time, the radius of the mycelium toward the yeast (R2) and the radius of the mycelium in the control sample (R1) were measured. Inhibition of pathogen growth was calculated using the following formula:

Inhibitory activity (%) = (R1 − R2)/R1 × 100

### 2.3. Effect on Pathogen Spore Germination In Vitro

Yeast was cultured in YPG medium (20 g glucose, 20 g peptone, 10 g yeast extract per 1 L distilled water; pH 6.0 ± 0.2) at 28 °C for 48 h and then centrifuged at 10,000 rpm for 10 min before being resuspended in sterile Ringer’s solution. Then, 0.1 mL of live yeast cells (5 × 10^8^ cells per mL) and 0.1 mL of a 10-day suspension of spores of the test fungi (5 × 10^6^ spores per mL) in Ringer’s solution were transferred to tubes with 4.8 mL of Wort medium (15 g malt extract, 1 g peptone, 12.5 g maltose, 2.5 g glucose, 1 g NH_4_Cl, and 1 g K_2_HPO_4_ per 1 L distilled water; pH 4.8 ± 0.2). The medium in which physiological sodium chloride solution was added instead of yeast was the control. All samples were incubated at 28 °C at 150 rpm for 24 h on a rotary shaker. In vivo preparations were then made in a Thoma counting chamber and the number of germinating spores per hundred observed was counted.

### 2.4. Hydrolytic Enzymes Production

The ability of yeasts to produce and secrete lytic enzymes was tested qualitatively (for protease, cellulase, and amylase) in solid media containing the respective substrates and quantitatively (for chitinase and β-1,3-glucanase) in liquid media with cell wall preparations (CWPs) of the tested fungi as enzyme inducers.

#### 2.4.1. Determination of Enzymes by the Qualitative Method

Proteolytic activity was evaluated in Petri dishes using skimmed milk agar (28 g skim milk powder, 2.5 g yeast extract, 5 g tryptone, 1 g dextrose, and 15 g agar per 1 L of distilled water; pH 7.0 ± 0.2). The medium was inoculated using the linear streak method with a loop of 2-day-old yeast culture and incubated at 28 °C for 7 days. Proteinase K (A&A Biotechnology, Gdansk, Poland) and sterile water were used as positive and negative controls, respectively. The presence of distinct zones of substrate discoloration determined proteolytic activity.

The cellulolytic activity was evaluated in Petri dishes using CMC agar (10 g yeast extract, 20 g peptone, 10 g carboxymethylcellulose (CMC) sodium salt, and 20 g agar per 1 L of distilled water, pH 6.0 ± 0.2). The medium was inoculated using the streaking method with a loop of 2-day-old yeast culture and incubated at 28 °C for 5 days. After this, the Petri dishes were stained with 0.1% Congo Red solution for 30 min and then washed with 1 M NaCl solution for 15 min. The presence of distinct zones of substrate discoloration determined cellulolytic activity.

The amylolytic activity was evaluated in Petri dishes using an agar medium supplemented with starch (20 g meat peptone, 20 g starch, 10 g yeast extract, and 12 g agar; pH 6.0 ± 0.2). The medium was inoculated using the streaking method with a loop of 2-day-old yeast culture and incubated at 28 °C for 5 days. After this time, the amylase plates were covered with Lugol’s solution, which was drained after 3 min of exposure, and the Petri dishes were left for at least 30 min. The clear zones of discoloration on the purple substrate determined amylolytic activity.

#### 2.4.2. Determination of Enzymes by the Quantitative Method

Tested yeast (0.5 mL suspension, 5 × 10^8^ cells per mL) was cultured in a shaking incubator (10,000 rpm) for 5 days at 28 °C in 30 mL of medium containing 3 g yeast extract, 5 g KH_2_PO_4_, 5 g (NH_4_)_2_SO_4_, 2.5 g CWP of *A. parasiticus* KKP731, and 2.5 g CWP of *A. ochraceus* KKP439 per 1 L distilled water. CWPs were prepared in accordance with the methods given by Chan and Tian [22]. Culture filters were taken from each flask and centrifuged at 10,000 rpm for 15 min. According to the previously described methodology [15], supernatants were used to analyze β-1,3-glucanase, chitinase, and protein content. The activities of chitinase and β-1,3-glucanase were evaluated by measuring the reducing sugars released from colloidal chitin and laminarin (Sigma-Aldrich, Poznań, Poland). One unit (U) of activity of each enzyme was defined as the amount of enzyme that yielded 1 μmol of glucose equivalent per milligram of protein per minute.

### 2.5. Ability to Survive in Different Environmental Conditions

The capacity of yeast to survive under different environmental conditions (including stress) was evaluated in Petri dishes for the following characteristics: temperature (4, 14, 18, 22, 30, 32, 37 °C), pH (4, 5, 6, 7, 9, and 11), and salinity (0, 2, 4, 6, 8, 10, 12, 14% NaCl). The 2-day yeast cultures in YPG medium were diluted to OD_600_ = 0.5. In each test, 0.1 mL of yeast suspension was inoculated into YPD media (10 g yeast extract, 20 g peptone, 20 g glucose, 20 g agar per 1 L distilled water; pH 6.0 ± 0.2) and incubated for 5 days. In the first test, the yeast was incubated at different temperatures. In the next experiment, YPD medium with a different pH was used, and the growth of the tested strains was evaluated under optimal temperature conditions of 28 °C. In another test, medium with different salt concentrations was used, and the Petri dishes were incubated at 28 °C.

### 2.6. Hydrogen Sulfide (H_2_S) Production

To test the ability to produce H_2_S, the methodology reported by Mamun-Or-Rashid et al. [23] was used. Yeast was inoculated using the streaking method with a loop of 2-day-old yeast culture on Bismuth Sulfite Agar (BSA) (Pol-Aura, Morąg, Poland) and Kligler Iron Agar (KIA) (Pol-Aura, Morąg, Poland) and incubated at 30 °C for 3 days. The brown or black color of the colonies on the BSA plates and the blackening of the KIA medium along the inoculation line indicated hydrogen sulfide production.

### 2.7. Biogenic Amines Determination

Determination of the tested yeast’s ability to produce biogenic amines was performed according to the methods presented by Aslankoohi et al. [24]. YPD medium was prepared with bromocresol red (0.006%) addition and supplemented with an amino acid mixture at a 1% total mass concentration. The amino acid mixture was composed of equal amounts of arginine, leucine, lysine, histidine, phenylalanine, tyrosine, and tryptophan. After the sterile medium solidified, Petri dishes were inoculated with 0.1 mL of 2-day-old yeast culture diluted to OD600 = 0.5 and then incubated at 28 °C for 72 h. The medium without added amino acids was the control of the experiment. A purple envelope around the colony evidences the production of biogenic amines.

### 2.8. Determination of Mycotoxin Content in Model Bread

The experiment determined the effect of yeast inoculum on the content of AFs and OTA in a model bread baked with the addition of flour from wheat grain contaminated with the mycelium of *A. parasiticus* KKP731 and the mycelium of *A. ochraceus* KKP439.

To contaminate the grains, 300 g of wheat and 10 mL of distilled water were added to a 1000 mL flask and autoclaved at 121 °C for 21 min. After cooling, the flask was inoculated with the mycelial fragments of two pathogens: *A. parasiticus* KKP731 and *A. ochraceus* KKP439. The flasks were left at room temperature for 15 days. After this time, the grain was evenly distributed and dried in a fume hood to a moisture content of 15%. Next, it was grounded in a laboratory grinder (IKA A11 basic, IKA-Werke GmbH & Co. KG, Staufen, Germany) and divided into two grinding fractions: flour and bran.

Baking was carried out according to the methodology of Escriva et al. [7,25] with some modifications, as described below. A loaf of bread was made from 100 g of flour (90 g of wheat flour type 750 and 10 g of contaminated wheat flour), 60 mL of warm water, 7 g of fresh baker’s yeast, 3 g of sugar, 2 g of salt, and 2% (*v*/*v*) addition of the test yeast *A. pullulans* PP3 and *S. podzolicus* D10 suspension at a density of 5 × 10^8^ cfu/mL. The control was the same dough without the addition of the test yeast suspension. The ingredients were mixed, and a dough was formed and allowed to rest for 3 h at 35 °C. Baking was carried out at 200 °C for about 20 min. The baked loaves were cooled under a sterilized laminar chamber. Then, they were cut into slices, placed on trays, and dried in an oven at 100 °C for about 2 h. The dried bread was ground in a laboratory grinder.

For the quantification of AFs and OTA, immunoenzymatic tests in a competitive format were carried out according to the attached instructions for test kits (RIDASCREEN^®^ Aflatoxin Total, RIDASCREEN^®^ Ochratoxin A 30/15, R-Biopharm, Darmstadt, Germany) using a microplate reader (Multimode Reader, Synergy HTX, BioTek, Winooski, VA, USA). Mycotoxin concentration was calculated from a standard curve determined from the absorbance of standards provided by the manufacturer.

### 2.9. Statistical Analysis

All experiments were made in triplicate, and the results are presented as means ± SEM (the standard error of the mean). Data obtained were analyzed statistically using Statistica (version 13.3, StatSoft, Cracow, Poland). A one-way analysis of variance (ANOVA) was performed, and the significance of differences between group means was evaluated using Tukey’s post hoc test. Statistical hypotheses were verified at the significance level of *p* < 0.05.

## 3. Results

### 3.1. Confrontation Assay

**Figure 1:**
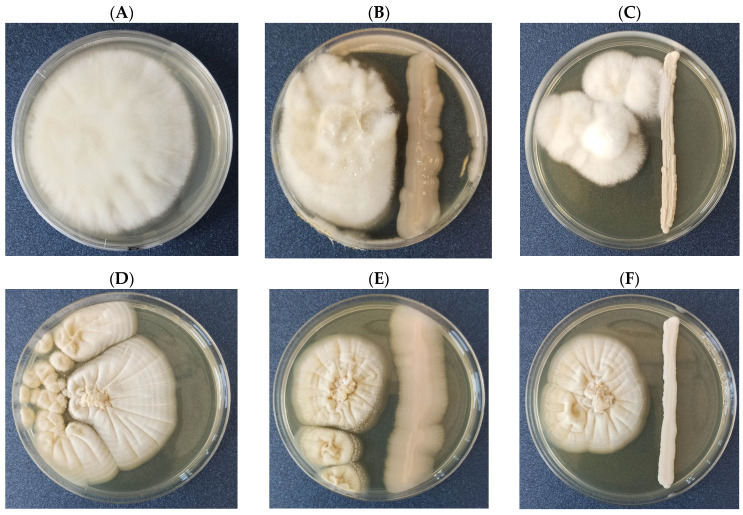
Mycelial growth inhibition of *A. parasiticus* KKP731 and *A. ochraceus* KKP439 by yeast isolates on Wort agar at 28 °C for 7 days: (**A**) *A. parasiticus* as a control; (**B**) a dual culture of *A. parasiticus* and *A. pullulans* PP3; (**C**) a dual culture of *A. parasiticus* and *S. podzolicus* D10; (**D**) *A. ochraceus* as a control; (**E**) a dual culture of *A. ochraceus* and *A. pullulans* PP3; (**F**) a dual culture of *A. ochraceus* and *S. podzolicus* D10.

Of the 51 yeast strains evaluated in the confrontation assay for their antagonistic activity against *A. parasiticus* KKP731 and *A. ochraceus* KKP439, only two strains demonstrated growth inhibition of both fungal pathogens (Figure 1). Strain PP3 isolated from cereals and identified as *Aureobasidium pullulans* inhibited the growth of *A. parasiticus* KKP731 and *A. ochraceus* KKP439 by 41.21 ± 2.91% and 53.64 ± 2.15%, respectively. Strain D10 from Symbio Bank (identified as *Saitozyma podzolicus* (homotypic synonym: *Cryptococcus podzolicus*)) inhibited the growth of *A. parasiticus* KKP731 by 43.25 ± 1.44% and *A. ochraceus* by KKP439 45.38 ± 2.60%. The other yeast strains tested in this study showed minimal or no growth inhibition of both pathogens.

### 3.2. Effect on Pathogen Spore Germination In Vitro

**Figure 2:**
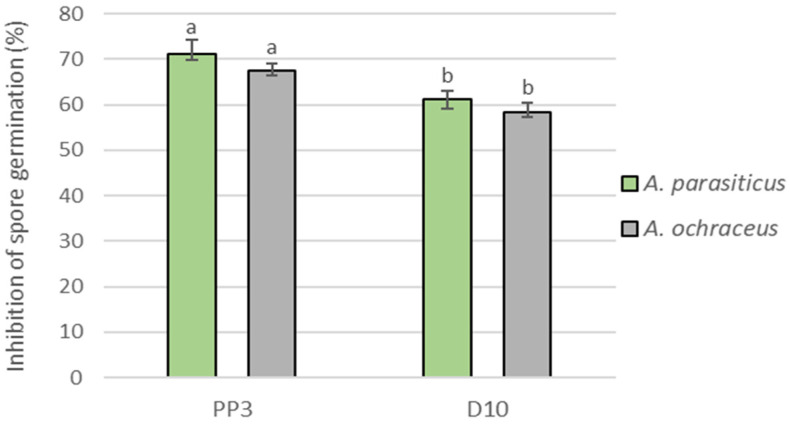
Inhibition of *A. parasiticus* and *A. ochraceus* spore germination by the yeasts *A. pullulans* PP3 and *S. podzolicus* D10 in Wort medium at 28 °C for 24 h. The error bar represents the standard error. Values marked with the same letters do not differ significantly at *p* < 0.05 (Tukey’s post hoc test).

After 24 h of yeast and fungi co-culture on a rotary shaker, effective inhibition of the spore germination of both tested pathogens was found (Figure 2). The more effective yeasts in this experiment were *A. pullulans* PP3. They inhibited they spore germination of *A. parasiticus* KKP731 by 71.22 ± 3.09% and *A. ochraceus* KKP439 by 67.52 ± 1.48%. *S. podzolicus* D10 inhibited the spore germination of *A. parasiticus* KKP731 by 61.15 ± 1.84% and *A. ochraceus* KKP439 by 58.39 ± 2.02%.

### 3.3. Hydrolytic Enzymes Production

The results of the enzymatic activity assays of the tested yeasts are shown in Table 1. For qualitative tests on hydrolytic enzyme production, yeast *A. pullulans* PP3 was found to produce proteases, cellulases, and amylases, while *S. podzolicus* D10 exclusively produced amylases. For quantitative tests in the medium using CWPs of *A. parasiticus* KKP731 and *A. ochraceus* KKP439 as the carbon source, *A. pullulans* PP3 was found to produce higher amounts of chitinase (1.79 ± 0.09 U/mg protein) and β-1,3-glucanase (7.26 ± 0.12 U/mg protein). *S. podzolicus* D10 yeast was shown to produce lower amounts of both enzymes, i.e., 0.66 ± 0.1 U/mg protein chitinase and 5.04 ± 0.09 U/mg protein β-1,3-glucanase. A common feature of both yeast species was higher production of β-1,3-glucanase than chitinase.

### 3.4. Ability to Survive in Different Environmental Conditions

The tested yeast strains differed in their growing capacity under different environmental conditions (Table 2). Active growth of the yeast *A. pullulans* PP3 was found in the 4–30 °C range with a pH gradient of 4–11, while *S. podzolicus* D10 growth was observed at 18–30 °C and with a pH gradient of 4–9. Under these conditions, both strains retained unchanged colony appearance compared to the control. At a temperature of 37 °C, the tested yeast had no growth. Under saline conditions, the yeast *S. podzolicus* D10 grew to 4% NaCl, while *A. pullulans* PP3 showed growth of up to 12%. A change in colony morphology under NaCl was observed in the *A. pullulans* PP3 yeast, which decreased with increasing NaCl concentration in the culture medium.

### 3.5. Hydrogen Sulfide (H_2_S) Production

The study found no hydrogen sulfide production by the yeasts *A. pullulans* PP3 and *S. podzolicus* D10 on Kligler Iron Agar or Bismuth Sulfite Agar (Table 2).

### 3.6. Biogenic Amines Determination

In the study, the ability to produce biogenic amines was not found in the yeasts *A. pullulans* PP3 and *S. podzolicus* D10 (Table 2). It was only found that both yeast species turned the culture medium yellow as a result of glucose fermentation.

### 3.7. Determination of Mycotoxin Content in Model Bread

**Figure 3:**
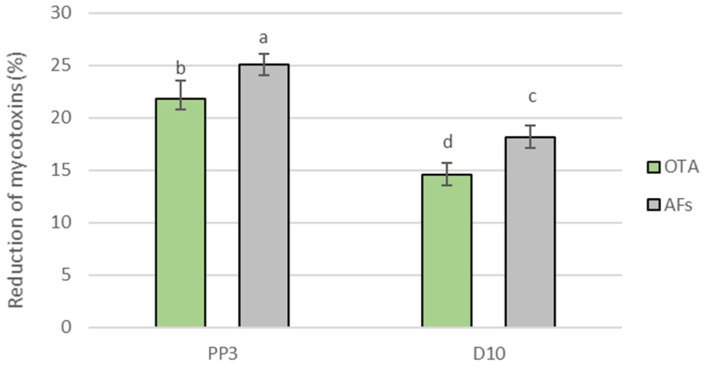
Reduction in ochratoxin A and total aflatoxins in model bread by the yeasts *A. parasiticus* PP3 and *S. podzolicus* D10. The error bar represents the standard error. Values marked with the same letters do not differ significantly at *p* < 0.05 (Tukey’s post hoc test).

It was found that the addition of the tested yeast inoculum to the dough resulted in reduced OTA and AF content in the model bread (Figure 3). It was also found that, of the two mycotoxins, both yeast strains reduced higher amounts of AFs. The yeast *A. pullulans* PP3 reduced both toxins (OTA in 21.80 ± 1.77% and AFs in 25.02 ± 1.06%) more effectively than the yeast *S. podzolicus* D10 (OTA in 14.55 ± 1.15% and AFs in 18.10 ± 1.13%).

## 4. Discussion

Since pathogens and antagonistic yeasts need nutrients and space for colonization and growth, many authors indicate competition for nutrients and space as one of the primary mechanisms of yeast action [26,27,28]. These two kinds of competition are generally considered together without giving each an appropriate level of importance. Biological control agents reduce pathogen population levels by depleting food sources because they are often more efficient at nutrient uptake than pathogens [26]. Competition for nutrients and living space requires the antagonist to multiply rapidly at low nutrient concentrations and better adapt to environmental conditions than the pathogen [13]. Yeast prevents the growth of undesirable microorganisms by rapidly depleting glucose, sucrose, or fructose from the medium [14]. Yeast has a high reproductive potential and thus shows the ability to quickly colonize the surface of crop plants, especially in areas of injury and tissue disruption resulting from mechanical damage caused during cultivation and harvesting [13]. Some microorganisms, including yeast, also form extracellular polysaccharide capsules that make it easier for them to adhere, for example, to the surface of fruits [26]. The study presented in this paper used a dual culture assay of the yeasts *A. pullulans* PP3 and *S. podzolicus* D10 with fungi to select growth inhibitors of *A. parasiticus* and *A. ochraceus*. Both isolates effectively reduced the two pathogens’ mycelial growth (above 40%) in Petri dishes. Thus, they confirmed effective competition with the pathogen for space or nutrients. These yeast species have already been studied for their antagonistic effects on various pathogens [3,29,30], with *A. pullulans* being more extensively studied and described in the literature [31,32,33]. Previous studies have also demonstrated the effectiveness of two wild yeasts, *A. pullulans* PP4 and *A. pullulans* ZD1, in limiting the growth of *A. flavus* in vitro and in vivo on tomato fruit [3].

Another biocontrol mechanism is the inhibition of fungal spore germination, demonstrated for the yeasts tested in this study against *A. parasiticus* and *A. ochraceus*. The effective action of the yeast species *A. pullulans* in inhibiting fungal spore germination has also been proven against other pathogens, including *A. falvus* [3], *Monilinia laxa* [34], *Monilinia fructicola*, and *Botrytis cinerea* [35]. Research studies in the literature suggest that various mechanisms are responsible for the ability of yeast to limit spore germination. Conidia require nutrients—sources of carbon, nitrogen, glucose, fructose, and iron—to germinate. By limiting the amount of available nutrients, antagonistic yeasts reduce the percentage of pathogen spore germination, consequently reducing spore follicle growth, infection, and necrosis and preventing pathogen expansion [26]. Furthermore, volatile organic compounds, enzymes, and antibiotic-like compounds also have a key role in this process [36]. Ando et al. [37] showed that the *Candida maltosa* NP9 yeast isolated from fermented food produced volatile compounds, i.e., isoamyl alcohol and isoamyl acetate, which inhibited the spore germination of 15 types of filamentous fungi (including several species of the genus *Aspergillus*).

Another important mechanism for the antagonistic action of yeast on filamentous fungi is lytic enzyme production, which leads to degradation of the fungal cell wall. The cell walls of fungi are dynamic structures necessary for cell viability, morphogenesis, and pathogenesis. They consist of 50–60% glucan (with a predominance of β-1,3-d-glucan), 10–20% chitin, and about 20–30% protein in the dry weight of the wall. The composition of the cell wall significantly impacts fungal ecology and is highly modulated in response to environmental conditions and imposed stresses [4,38]. The production of lytic enzymes by yeast is affected by various factors, including the strain used, conditions, and culture duration. Also important is the medium used, including the carbon sources used as inducers of enzyme biosynthesis. The use of various culture media often provides different results. For example, results obtained by Zhao et al. [29] showed that the β-glucan added to the culture medium was an inducer of β-1,3-glucanase production by the yeast *C. podzolicus*. In the study presented in this paper, the yeasts tested showed varying enzymatic activity, with *A. pullulans* being the better enzyme producer and showing higher chitinase and β-1,3-glucanase activity. Previous studies have shown similar activity in these enzymes for the yeast *A. pullulans* when isolated from soil. As in the presented study, the inducers of chitinase and β-1,3-glucanase production were the CWPs of the tested pathogen [3]. Analyses by other authors confirm the ability of this yeast species to produce several other enzymes, including cellulase, protease, amylase, pectinase, and lipase [39]. Similarly, enzymatic activity has been demonstrated for the yeast *C. podzolicus*, including the production of xylanase, amylase, pectinase, and protease [40].

Screening for the selection of an effective antagonistic microorganism requires not only understanding of its mechanisms of action but also testing of its growth and survival conditions [41]. An effective antagonist should have mechanisms for dealing with the many abiotic stresses to which it is exposed [42]. Many biocontrol agents exhibit significant stress tolerance and can adapt to low nutrient availability, high concentrations of various dissolved substances, variable pH, and high and low temperatures [41]. Greater capacity to grow than a pathogen and survival ability in harsh environments are important characteristics of biocontrol agents [43]. Many species of *Aspergillus* fungi can grow in environments poor in essential nutrients and thrive in a wide range of temperature (10–50 °C), pH (2–11), and salinity (0–34%) conditions [44]. In the work presented here, effective antagonists against selected species of the genus *Aspergillus* were sought, so it was appropriate to study various aspects of the polyextremotolerance of the tested yeast strains, including their tolerance to different temperatures, pH, and salinity. Of the two yeast species, only *A. pullulans* PP3 could grow actively under refrigerated conditions of 4 °C. This is a relevant feature of biocontrol because many fruits and vegetables are stored in such conditions. The yeast *A. pullulans* also showed a wider tolerance range to the medium’s pH (4–11) and salinity (0–12% NaCl). These properties vary depending on the strain tested. Other authors report that this species can thrive at concentrations of up to 18% NaCl [31]. Studies in the literature suggest that tolerance to high osmolarity may be associated with adaptation to other stress conditions (e.g., temperature stress, oxidative stress, heavy metal stress) [45], which can also play an important biocontrol role. The tolerance of *A. pullulans* to various ecological conditions allows it to inhabit multiple niches and ensures adaptability and survival [39].

Data from the literature show great potential for non-conventional yeast strains, not only as biocontrol agents in the crop field [27] but also in food technology [46]. The resistance to stress conditions in *A. pullulans* PP3 demonstrated in this study is a desirable trait for baker’s yeast. Yeasts used in baking encounter stressors during biomass preparation and dough fermentation, including heat stress, osmotic stress, salinity stress, ethanol stress, oxidative stress, or freeze–thaw stress [46]. Non-conventional yeast creates new opportunities in the modern baking industry by increasing the complexity of bread aromas [24]. Unfortunately, there is also the possibility that wild yeast will produce compounds that are disorderly in food production, affecting the taste or smell of the finished product; thus, proper testing should be conducted to select strains. The yeast tested in this study did not produce hydrogen sulfide, which is associated with an unpleasant taste and aroma. The production of this compound by yeast is an undesirable feature that reduces bread quality [47]. The study presented here also tested the safety of using yeast isolates in food technology by determining the possibility of producing biogenic amines (amino acid decarboxylation products). These compounds are dangerous because they act as neurotoxins when absorbed at high levels by the human body [48]. Another important characteristic that should be examined before using new isolates in food production is the capability to grow at 37 °C and above, eliminating concern for human safety. This is the most apparent virulence factor of human pathogens and one of the major risk factors when assessing potential biocontrol agent safety [31]. Preliminary safety tests of the studied isolates of *A. pullulans* PP3 and *S. podzolicus* D10 confirm that they do not produce biogenic amines and do not show the ability to survive at 37 °C.

Yeast can also contribute to product safety by acting as a biopreservative and reducing the concentration of mycotoxins in the food product. Such properties were demonstrated for *S. cerevisiae* RC008 and RC016, which inhibited the growth of the fungi *A. carbonarius* and *Fusarium graminearum* and reduced the production of OTA, zearalenone (ZEA), and deoxynivalenol (DON) [49]. Mozaffary et al. [20] also confirmed that the baker’s yeast *S. cerevisiae* reduced OTA by about 60% during bread dough fermentation. Chlebicz and Śliżewska [50] showed that six strains of the yeast *S. cerevisiae* effectively reduced the amounts of a mixture of fumonisin B1 and B2 (by 67–74%), AFB1 (by 65%), T-2 toxin (by 69%), ZEA (by 52%), and DON (by 22–43%). There are many examples in the literature that non-conventional yeasts can also be effective in reducing mycotoxins in many food products. Previous studies confirmed an effective reduction in the content of DON (by 16.4–33.4%), nivalenol (NIV) (by 18.5–36.2%), and ZEA (by 14.3–35.4%) in bread prepared with the addition of the following yeast inoculums: *Candida shehatae*, *Candida fluviatilis*, *Meyerozyma guilliermondii*, *Cyberlindnera saturnus*, and *Rhodotorula glutinis* [16]. Repečkienė et al. [17] proved the efficacy of the yeasts *Kluyveromyces marxianus*, *Geothrix fermentans*, *Metschnikowia pulcherrima*, and *S. cerevisiae* in the elimination of AFs, ZEA, and DON from wheat flour and feed. Taheur et al. [18] confirmed the ability of *Kazakhstania servazzii* yeast to remove OTA, AFB1, and ZEA from milk by 74%, 62%, and 95%, respectively. In this study, bread baked with the addition of flour from grain contaminated with *A. parasiticus* KKP731 and *A. ochraceus* KKP439 mycelium (for natural mycotoxin production) and an inoculum of the non-conventional yeast strains *A. pullulans* PP3 and *S. podzolicus* D10 showed a reduction in OTA and AFs compared to control bread. The results of microbial decontamination are difficult to compare because it is a highly variable process that depends on the strain used, the physiological state of the cells, environmental conditions, and the initial concentration of the toxin. There are reports in the literature confirming the ability of the tested yeast species to reduce mycotoxins. Wei et al. [51] confirmed the ability of the yeast *C. podzolicus* Y3 to completely eliminate OTA (1 μg/mL) in PM medium within 5 days and in grape juice medium within 3 days. This mycotoxin was effectively eliminated to non-toxic ochratoxin α (OTα). Furthermore, it has been shown that *C. podzolicus* Y3, together with OTA, also degrades citrinin (CIT). The ability to reduce the amount of toxins has also been demonstrated for the yeast *A. pullulans*, such as in the reduction in the amount of OTA in grapes [52]. Tsitsigiannis et al. [53] reported that *A. pullulans* is able to control the fungi *A. carbonarius*, *A. niger*, and *Penicillium expansum* and the mycotoxins they produce, i.e., OTA and patulin, respectively. The mechanism responsible for reducing the concentration of mycotoxins through the use of yeast was not investigated in the study presented here, but there are reports in the literature that such mechanisms could be bioadsorption, biodegradation, or inhibition of toxin production [54]. It has also been shown that the ability to remove toxins can be increased by using various additional processes, such as heat treatment of the tested microorganism or lowering the pH of the medium [55].

## 5. Conclusions

There are a growing number of studies worldwide that highlight the potential of non-conventional yeasts in the development of new bakery products. The presented study shows that selected yeasts can be effective biocontrol agents for fungal pathogens, exhibiting different mechanisms of action. It also suggested the possibility of using them in food technology to improve the safety of a finished product such as bread. According to the preliminary results obtained in this study, the selected yeast strains exhibited several characteristics of interest to the baking industry, such as thermotolerance, halotolerance, and osmotolerance, among others. The selected isolates also represent a promising ingredient for new starter cultures aimed at reducing the content of mycotoxins that are dangerous to health, as highlighted by the results presented in this study. However, further research is needed to fine-tune different production processes and verify that the selected strains possess the Generally Recognized as Safe (GRAS) and Qualified Presumption of Safety (QPS) status to be used in the food industry.

## Figures and Tables

**Table 1:** Enzymatic activities of the yeasts *A. pullulans* PP3 and *S. podzolicus* D10.

Yeast	Qualitative Determination (±) *			Quantitative Determination (U/mg Protein)	
	Protease	Cellulase	Amylase	Chitinase	β-1,3-Glucanase
*Aureobasidium pullulans* PP3	+	+	+	1.79 ± 0.09	7.26 ± 0.12
*Saitozyma podzolicus* D10	−	−	+	0.66 ± 0.1	5.04 ± 0.09

* + indicates positive reaction; − indicates negative reaction.

**Table 2:** Preliminary characteristics of the yeasts *A. pullulans* PP3 and *S. podzolicus* D10.

			Yeast Strain	
			*A. pullulans *PP3	*S. podzolicus* D10
Growth conditions	Temperature (°C)	4	+	−
		14	+	−
		18	+	+
		22	+	+
		30	+	+
		32	−	−
		37	−	−
	pH	4	+	+
		5	+	+
		6	+	+
		7	+	+
		9	+	+
		11	+	−
	NaCl (%)	0	+	+
		2	+	+
		4	+	+
		6	+	−
		8	+	−
		10	+	−
		12	+	−
		14	−	−
H_2_S production			−	−
Biogenic amine production			−	−

+ growth; − absence of growth.

## Data Availability

The data presented in this study are available on request from the corresponding author.
